# Assessment on clinical value of prostate health index in the diagnosis of prostate cancer

**DOI:** 10.1002/cam4.2376

**Published:** 2019-07-17

**Authors:** Guangying Zhang, Yanyan Li, Chao Li, Na Li, Zhanzhan Li, Qin Zhou

**Affiliations:** ^1^ Department of Oncology Xiangya Hospital, Central South University Changsha China; ^2^ Department of Outpatient Xiangya Hospital, Central South University Changsha China

**Keywords:** prostate cancer, prostate health index, sensitivity, specificity

## Abstract

In this study, we performed a comprehensive estimation and assessment for the clinical value of prostate health index (PHI) in diagnosing prostate cancer. Using the bivariate mixed‐effect model, we calculated the following parameters and their 95% confidence internals (CIs), including sensitivity, specificity, positive likelihood ratio, negative likelihood ratio, diagnostic odds ratio and symmetric receiver operator characteristic. Twenty eligible studies with a total number of 5543 subjects were included in the final analysis. The estimated sensitivity was 0.75 (95% CI: 0.70‐0.79) and the specificity was 0.69 (95% CI: 0.58‐0.83). The pooled area under the curve was 0.78 (95% CI: 0.74‐0.81). The combined positive likelihood ratio was 2.45 (95% CI: 2.19‐2.73) and the negative likelihood ratio was 0.36 (95% CI: 0.31‐0.43). The diagnostic odds ratio was 6.73 (95% CI: 5.38‐8.44). The posttest probability was 40% under the present positive likelihood ratio of 2.45. It seems there was no significant difference between Asian population and Caucasian population population in sensitivity and specificity. But the overlap of AUC 95% CI indicated that the diagnostic accuracy of PHI was slightly higher in the Asian population population setting than that in the Caucasian population population population (0.83 vs 0.76). Similarly, there was also overlap in AUC 95% CI, which suggested that sample size may be one of heterogeneity source. The PHI has a moderate diagnostic accuracy for detecting prostate cancer. The discrimination ability of PHI is slightly prior to free/total prostate‐specific antigen. It seems that ethnicity has an influence on the clinical value of PHI in the diagnostic of prostate cancer.

## 1 INTRODUCTION

Prostate cancer is a common and major cancer of the male genitourinary system and ranks the second among male common malignancy around the world.^1^, ^2^ The incidence of prostate cancer in the USA has surpassed that of lung cancer as the first cancer to harm men's health. It was estimated by American cancer society that there were about 164 690 new cases of prostate cancer in the USA in 2018, and 29 430 cases died from this disease.^3^ About 4.5 million new cases of prostate cancer are confirmed in Europe each year. The ratio of prostate cancer was 21.8% among all male cancers and accounted for 10% of cancer deaths.^4^ The incidence of prostate cancer is lower in Asian population countries than that in Europe and Americas. Prostate cancer's incidence is also relatively low in China. However, as the proportion of the population aging and lifestyle changes in China, it is growing faster than other malignant tumors.^5^ According to the latest data from the national cancer center in 2008, prostate cancer has surpassed bladder cancer and becomes the most common cancer in the male genitourtinary system.^6^ In 2009, its incidence reached 8 out of 100 000, ranking the fifth among male malignant tumors and the mortality rate reached 4.19 out of 100 000, ranking the ninth among all male malignant tumors.^7^ Early identification and treatment for patients with prostate cancer seem to be particularly important.

It is of great importance for cancer patients to receive early screening and diagnostic because early identification may hugely affect treatment and prognosis. Since the US food and drug administration approved the usage of serum prostate‐specific antigen (PSA), PSA had become a widespread practice in detecting prostate cancer.^8^ However, there exist some disputes in the diagnosis accuracy of PSA because of some potential factors such as benign prostatic hyperplasia, inflammation, age, and drug.^9^, ^10^, ^11^ Therefore, an accurate diagnostic marker for prostate cancer can help clinicians and patients to better diagnose and treat the disease. In recent years, many researchers are looking for other highly specific diagnostic markers for prostate cancer.^12^ The prostate health index (PHI) is calculated using the following index: total PSA, free PSA, and pro‐PSA. The FDA recommended the PSA could be considered as an early diagnostic biomarker of prostate cancer because a lot of prospective observational studies from the USA and Europe have suggested that PHI has the highest sensitivity and specificity for prostate cancer.^11^ Some studies have assessed the diagnostic ability of PHI for prostate cancer. In the present study, we systematically searched the literature and performed a comprehensive estimation and assessment for PHI in detecting prostate cancer.

## 2 MATERIALS AND METHODS

We performed this study by following the Preferred Reporting Items for Systematic Reviews and Meta‐Analyses guideline (Supplementary Material S1).

### 2.1 Literature search

We performed a systematical search in the several commonly used databases: China national knowledge infrastructure, and Wanfang, Embase Web of Science and PubMed, with the updated data of 10 April 2019. The following search words were used: ‘prostatic neoplasms’ OR ‘prostate cancer’ OR ‘prostate tumor’, screening, sensitivity, specificity, receiver operating characteristic curve, ROC, diagnostic OR diagnosis, PHI OR PHI. The search language was restricted in Chinese and English. To obtain potential relevant study, we also checked the references lists of articles and reviews.

### 2.2 Criteria for inclusion and exclusion

The included studies should meet the followed criteria: (a) topic about the diagnostic accuracy assessment of PHI for prostate cancer; (b) cancer diagnosis was confirmed by pathology gold criteria; (c) sufficient data (TP: true positive, FP: false positive, FN: false negative, TN: true negative) can be extracted for pooling. Exclusion criteria: (a) For republicated data and study, the latest study was used; (b) study cannot obtain effective data or other information; (c) irrelevant study and topic; (d) letter, reviews, comments, animal study were also excluded. Two investigators independently performed the screening process by scanning title, abstract and full‐test. We resolved the disagreements by consensus.

### 2.3 Data extraction

Two researchers independently extracted the data. Disagreements were resolved by consensus. We mainly extracted the following data for each study: the name of first author, the year of publication, country, study design (retrospective vs prospective), age (mean age or median age), gold standard, PHI cut‐off value, sample size, sensitivity, specificity, and four folds data including TP, FP, FN and TN.

### 2.4 Quality assessment of included study

We used the QUADAS‐2 (quality assessment of diagnostic accuracy studies‐2) tool to perform the quality assessment.^13^, ^14^ This scale consists of four domains: patients’ selection, index test, references standard and flow and timing. Every domain included two subdomains: risk of bias and concerns regarding applicability. For risk of bias, we can judge yes, no or unclear risk for each item, any of several items was judged as no, then we can give a high‐risk judgment. For concern regarding applicability, we can give low concern, high concern and or unclear concern based on the study. We used risk of bias and applicability concerns graph to present the results of quality assessment.

### 2.5 Statistical analysis

We used the bivariate mixed‐effect model to pool the following index and their 95% confidence internals (CIs)^15^: sensitivity, specificity, positive likelihood ratio (PLR), negative likelihood ratio (NLR), diagnostic odds ratio (DOR), and symmetric receiver operator characteristic (AUC).^16^ For sensitivity, specificity, and AUC, a value of 1.0 was considered as the highest diagnostic accuracy and AUC < 0.5 indicated a poor diagnostic accuracy.^17^, ^18^, ^19^ The heterogeneity within studies was assessed by using the Q test and *I* ^2^ statistic. *I* ^2^ > 50% and/or *P* < 0.05 indicated significant heterogeneity.^20^, ^21^ The random and fixed effect models would be selected based on whether the heterogeneity existed or not. Subgroup analysis was performed under the following factors: ethnicityity (Asian population vs Caucasian population population), study design (retrospective vs prospective), median sample size (>250 vs ≤250), and median age (60‐69). We used the Fagan's nomogram to assess the relationship between pretest probability and posttest probability.^22^ The asymmetry of Deeks plot was used to detect the publication bias.^23^ The sensitivity analysis was performed by deleting study with sample size <100, study with age >70 and abnormal cut‐off value. We performed all statistical analyses using Stata 14.0 software (Corp, College Station, TX) and RevMan5 *P* < 0.05 indicated statistically significant.

## 3 RESULTS

### 3.1 Study selection and general characteristics

We obtained 687 records from the initial search. Four hundred and seventeen records were ready for further screening after the duplicates were excluded. We further excluded 339 records via scanning the title and abstract and left 78 records for full‐text assessment. Fifty‐eight records were excluded including 42 records with unrelated topics and diagnostic values, 10 records with insufficient data, six reviews, comments, letter and meeting abstract. Finally, 20 eligible studies were included in the final analysis.^24^, ^25^, ^26^, ^27^, ^28^, ^29^, ^30^, ^31^, ^32^, ^33^, ^34^, ^35^, ^36^, ^37^, ^38^, ^39^, ^40^, ^41^, ^42^, ^43^ The Supplementary Material S2 presented the general features of included studies in the present study. The total sample size of 20 studies was 5543 including 2258 cases and 3285 controls. The largest and smallest sample sizes were 892 and 50, respectively. These studies were published from 2011 to 2018. Eight studies were from Asian population countries including Japan for one and seven for China. Twelve studies were from Caucasian including Spain for one, USA for three, Italy for seven and France for one. Of all studies, five studies were based on retrospective design and 15 studies were prospective design. The mean/median age of included studies ranged from 60 to 71.5. Only one study included study populations whose age was more than 70 years old. The sensitivity was from 0.60 to 0.90 and the specificity was from 0.43 to 0.80. Most of the optimal cut‐off values fallen into the range between 40 and 50. The cut‐off values of two studies were about 30 and of one study were more than 50.

### 3.2 Assessment of quality

**Figure 1:**
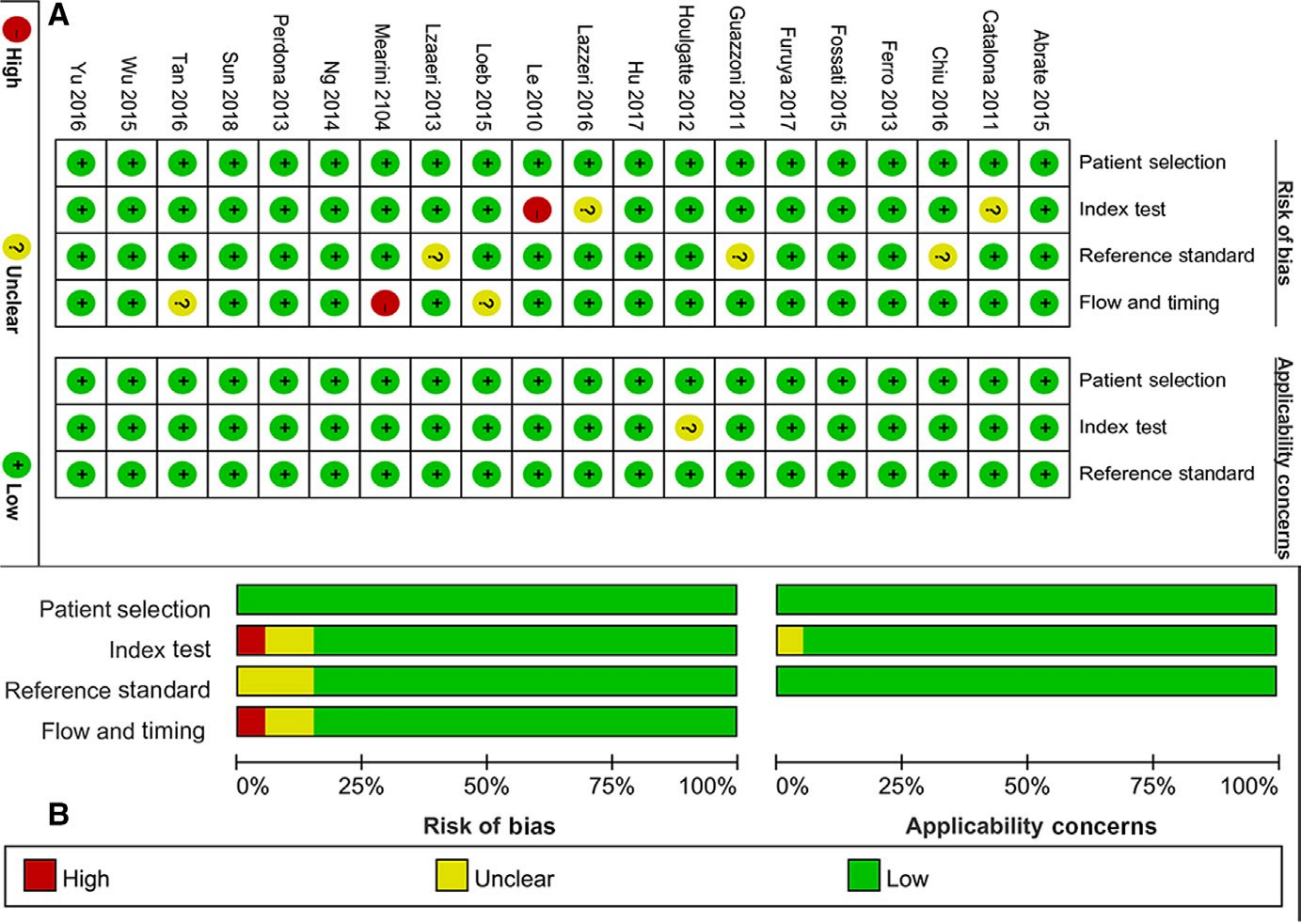
Quality assessment of the included studies: (A) judgements about each domain for each included study; (B) judgements about each domain presented as percentages

The Figure 1A and Figure 1B presented the authors' quality assessment of each study. All studies have no high risk of bias in patient selection, and no high risk concerned in patient's selection and reference standard. Generally speaking, two studies were judged as unclear risk of bias in index test. One study in index test and one study in flow and timing were considered as high risk of bias. Three studies have unclear risk of bias in references standard. Two studies also were unclear involved in flow and timing. The ratio of high‐risk bias is 10%, and ratio of unclear risk bias is 12.5% for each subitem. In statistical terms, the overall quality is quite high.

### 3.3 Pooled results

**Figure 2:**
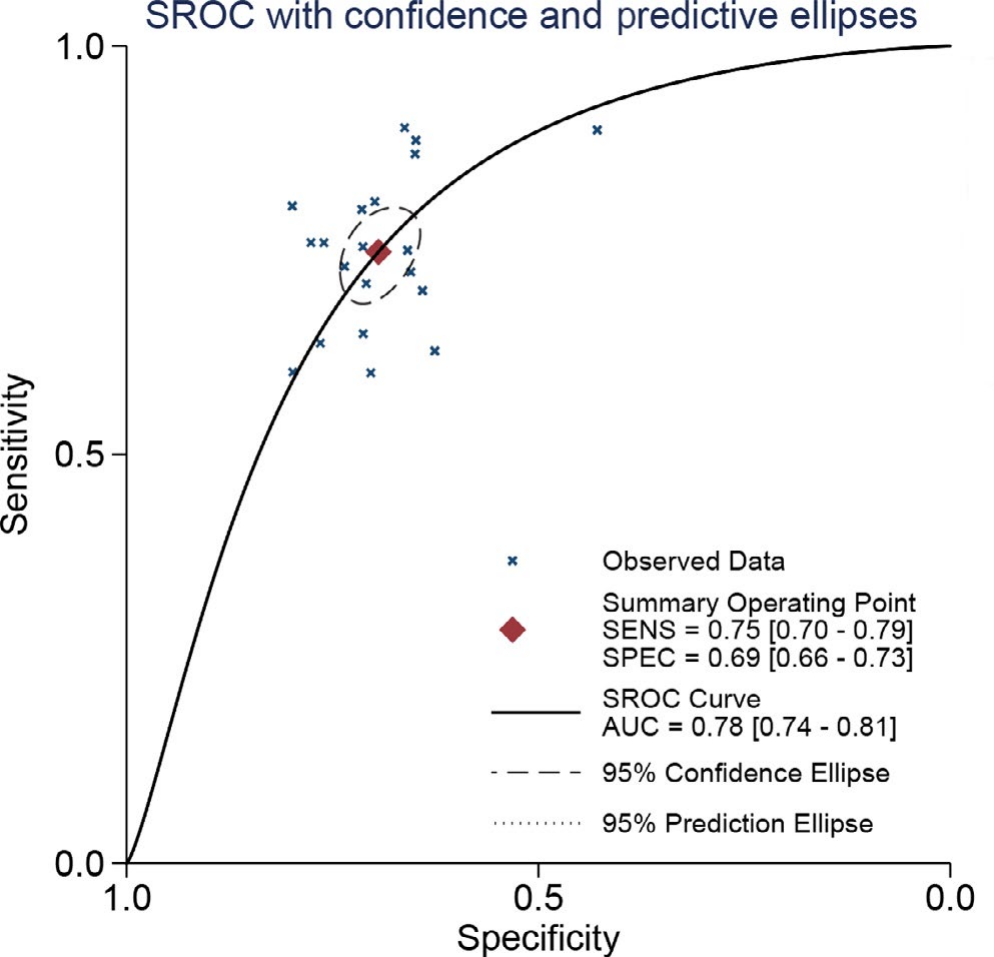
The SROC curve of prostate health index for prostate cancer

**Figure 3:**
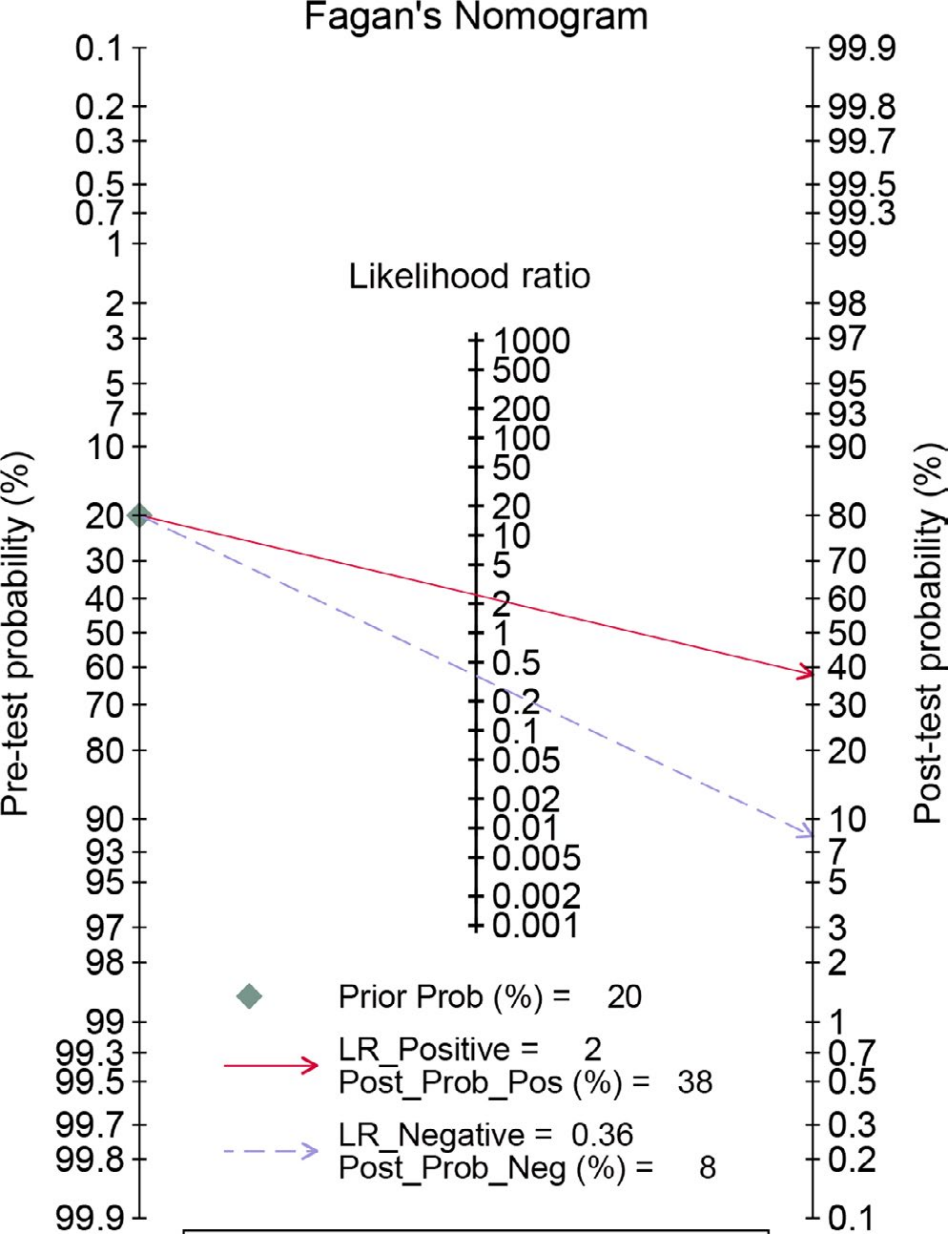
Fagan diagram assessing the overall diagnostic value of prostate health index for prostate cancer

There was high heterogeneity within studies (*I* ^2^ = 79.36% and 80.39%, respectively, and *P* < 0.05). The results from random‐effect models indicated that the combined sensitivity was 0.75 (95% CI: 0.70‐0.79) and the combined specificity was 0.69 (95% CI: 0.58‐0.83). The pooled AUC was 0.78 (95% CI: 0.74‐0.81, Figure 2). These results indicated that the PHI has a moderate diagnostic ability for prostate cancer. The combined PLR and NLR were 2.45 (95% CI: 2.19‐2.73) and 0.36 (95% CI: 0.31‐0.43), respectively. The DOR was 6.73 (95% CI: 5.38‐8.44). The Figure 3 presented the Fagan's nomogram. If the pretest probability was 20%, the posttest probability achieved a value of 40% based on the pooled PLR and the pretest probability of 20%.

We also performed the subgroup analysis in different ethnicityity (Asian population vs Caucasian population population), study design (prospective vs retrospective), and sample size (>250 vs ≤250). For Asian population population, the estimated sensitivity and specificity were 0.80 (95% CI: 0.75‐0.84) and 0.71, respectively (95% CI: 0.67‐0.75). The estimated AUC is 0.83 (95% CI: 0.79‐0.86). For Caucasian population population, the pooled sensitivity and specificity were 0.72 (95% CI: 0.66‐0.78) and 0.68 (95% CI: 0.62‐0.73), respectively. The estimated AUC is 0.76 (95% CI: 0.72‐0.79). It seems there were no significant differences in sensitivity and specificity. But the nonoverlap of AUC 95% CI indicated the diagnostic accuracy of PHI was slightly higher for Asian population population setting than that in the Caucasian population population population setting. For study design, no significant differences were observed in sensitivity (0.74 vs 0.78), specificity (0.69 vs 0.70), AUC (0.77 vs 0.80), PLR (2.39 vs 2.59), NLR (0.38 vs 0.31), and DOR (6.23 vs 8.25). The 95% CIs are presented in the Table 1. For sample size >250 group, the specificity and specificity were 0.74 (95% CI: 0.68‐0.79) and 0.71 (95% CI: 0.65‐0.76), respectively. For ≤250 group, the sensitivity was 0.77 (95% CI: 0.69‐0.83) and specificity was 0.67 (95% CI: 0.64‐0.75). Similarly, there was also no overlap in AUC 95% CI, which suggested that sample size may be one of heterogeneity source. The Table 1 presented more details.

**Table 1:** Summary results of diagnostic performance of prostate health index for prostate cancer

Category	SEN [95% CI]	SPE [95% CI]	PLR [95% CI]	NLR [95% CI]	DOR [95% CI]	AUC [95% CI]
Overall	0.75 [0.70‐0.79]	0.69 [0.66‐0.73]	2.45 [2.19‐2.73]	0.36 [0.31‐0.43]	6.73 [5.38‐8.44]	0.78 [0.74‐0.81]
ethnicityity						
Asian population	0.80 [0.75‐0.84]	0.71 [0.67‐0.75]	2.76 [2.41‐3.15]	0.29 [0.23‐0.36]	9.65 [7.17‐12.99]	0.83 [0.79‐0.86]
Caucasian population population	0.72 [0.66‐0.78]	0.68 [0.62‐0.73]	2.24 [1.97‐2.55]	0.41 [0.34‐0.49]	5.47 [4.33‐6.93]	0.76 [0.72‐0.79]
Study design						
Prospective	0.74 [0.68‐0.78]	0.69 [0.65‐0.73]	2.39 [2.12‐2.68]	0.38 [0.33‐0.45]	6.23 [4.99‐7.77]	0.77 [0.73‐0.81]
Retrospective	0.78 [0.70‐0.84]	0.70 [0.64‐0.75]	2.59 [2.05‐3.28]	0.31 [0.22‐0.45]	8.25 [4.66‐14.59]	0.80 [0.76‐0.83]
Sample size						
>250	0.74 [0.68‐0.79]	0.71 [0.65‐0.76]	2.54 [2.16‐2.98]	0.37 [0.31‐0.45]	6.87 [5.22‐9.03]	0.79 [0.75‐0.82]
≤250	0.77 [0.69‐0.83]	0.67 [0.64‐0.70]	2.31 [2.04‐2.61]	0.35 [0.26‐0.47]	6.60 [4.43‐9.82]	0.69 [0.64‐0.73]
Sensitivity analysis						
Age within 60‐69	0.76 [0.71‐0.80]	0.70 [0.65‐0.75]	2.55 [2.22‐2.94]	0.34 [0.29‐0.41]	7.44 [5.89‐9.39]	0.80 [0.76‐0.83]
Deleting studies with sample size <100	0.75 [0.70‐0.79]	0.69 [0.66‐0.73]	2.44 [2.18‐2.73]	0.37 [0.31‐0.43]	6.68 [5.30‐8.43]	0.78 [0.74‐0.81]
Deleting study with age >70	0.74 [0.69‐0.78]	0.70 [0.66‐0.74]	2.46 [2.18‐277]	0.37 [0.32‐0.44]	6.60 [5.22‐8.34]	0.78 [0.74‐0.81]
Deleting cut‐off value >40 or >50	0.74 [0.68‐0.80]	0.69 [0.63‐0.74]	2.38 [2.03‐2.79]	0.38 [0.31‐0.46]	6.31 [4.76‐8.37]	0.77 [0.74‐0.81]

### 3.4 Sensitivity analysis and publication bias

**Figure 4:**
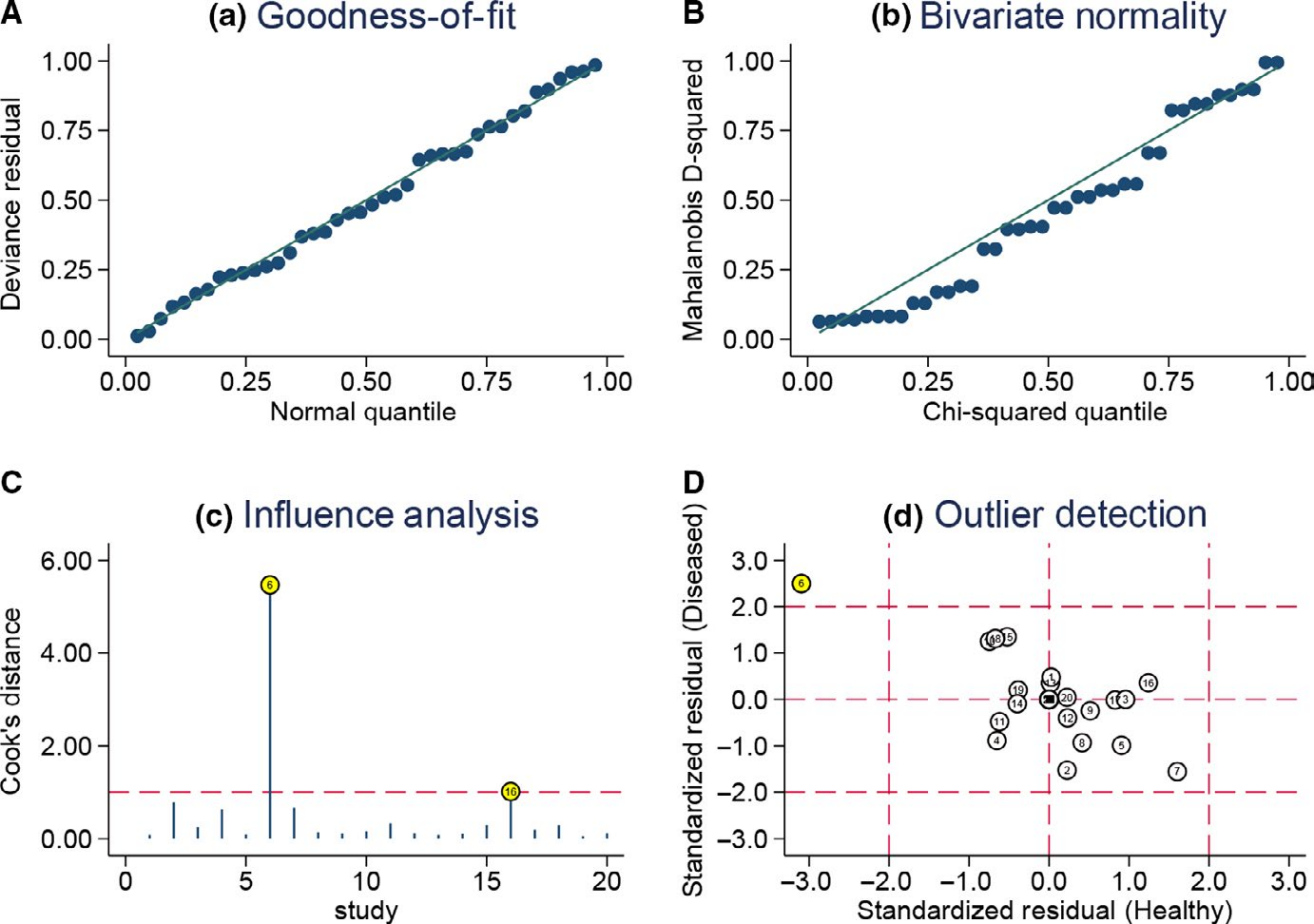
Sensitivity analyses: graphical depiction of residual based goodness‐of‐fit (A), bivariate normality (B), and influence (C) and outlier detection (D) analyses

**Figure 5:**
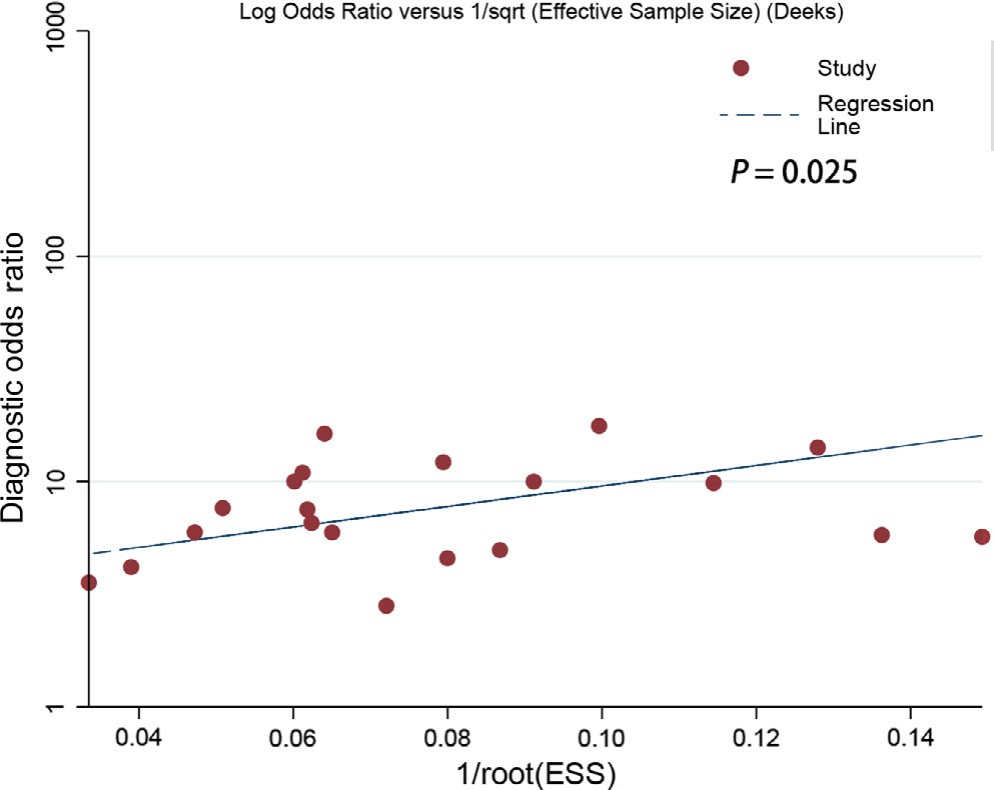
Deeks’ plot of Publication bias

We performed the sensitivity analysis via deleting some studies. Specifically, we performed pooled estimation via excluding age >70 or <60, sample size <100, and special cut‐off value. We also used the “modchk” method to perform the sensitivity analysis. The Figure 4 revealed that single study did not alter the final results. The influence analysis and outlier detection indicated that only two studies may have affected the results. The pooled results indicated that the diagnostic ability kept stable. The overall sensitivity, specificity, and AUC did not alter. The Table 1 gave the specific results. The Deeksplot (Figure 5) indicated that some studies slightly diverged from the regression line, which indicated that the publication bias may exist. The quantitative test result also gives some clues (*t* = 2.450, *P* = 0.025).

## 4 DISCUSSION

The present results with 20 studies indicated that the combined sensitivity was 75% and the specificity was 69% with an AUC of 0.78. The pooled results suggested that the PHI has a moderate diagnostic ability for detecting prostate cancer. With the high heterogeneity within studies, the subgroup analysis was performed. There seemed to be no significant differences for the diagnostic ability of PHI in different population setting, study design type, age, and cut‐off values. No overlap of confidence interval indicated the and population setting and sample size may be one of the potential heterogeneity sources.

The early diagnosis and screening of the prostate cancers is always a huge challenge. The diagnostic gold standard for prostate cancer is prostate biopsy. Previous studies also reported other methods such as digital rectal examination and transrectal ultrasonography. However, all these methods made patients feel embarrassed and uncomfortable because these methods were extremely invasive.^12^ The PHI is a comprehensive evaluation index that includes serum total PSA, free PSA and −2pro‐PSA (PHI was calculated referring to the following formula [−2]pro‐PSA/fPSA × √PSA).^44^ Catalona et al conducted a comparative research in a population with 892 men. They found that the diagnostic accuracy of PHI (AUC = 0.724) was superior to free PSA/total PSA (AUC = 0.670) in detecting Gleason 4 or greater prostate cancer among low‐grade and control population.^25^ In fact, researchers had raised doubt to the diagnostic accuracy of PSA for prostate cancer. It was reported that people were still diagnosed with prostate cancer even when the PSA level was under the cut‐off value. For men under 60 years old, the specificity was very high (0.98) but the sensitivity was quite low (0.18), which means that 82% of men would undergo unnecessary biopsy and treatment.^45^ Scattoni et al performed a head‐to‐head comparison of PHI and prostate cancer antigen 3 (PCA3) in 211 patients undergoing prostate biopsy. They reported that the PHI (0.7) was better than PCA3 (0.59), total/free PSA (0.56, 0.60). PHI showed optimal diagnostic accuracy in both initial setting and repeat setting. The present results found that PHI may be even better that this study (AUC = 0.78).^46^ In parallel with two studies above, Loeb et al performed a prospective study in 658 50‐year or older men and made comparisons among PSA. Free PSA, pro‐PSA and PHI. Of all these parameters, PHI had the highest diagnostic accuracy of prostate cancer. At the 0.90 of sensitivity cut point for PHI, 30% of patients avoided an unnecessary biopsy. And this value was 21.7% for free PSA.^34^ However, Perdona et al performed a prospective observational study in 160 men. They found %p2PSA (AUC = 0.68), PHI (AUC = 0.71) and PCA3 (AUC = 0.66) can give a good diagnostic ability for prostate cancer. The pairwise‐comparison indicated that there was no significant difference between PHI and PCA3 in the diagnosis of prostate cancer for men who underwent first prostate biopsy.^36^ Ferro et al also reported similar results. They found the diagnostic ability of PHI was similar to PCA3 and %p2PSA. No significant differences were observed for these three parameters. However, they are superior to free PSA, %free PSA, and p2PSA.^40^ Previous also study also assessed the clinical diagnostic value of free/total PSA ratios for prostate cancer using meta‐analysis. The combined sensitivity was 0.7 and the specificity was 0.55. The AUC was 0.76 and was near close to the present study results.^47^ These results suggested that PHI that combines serum total PSA, free PSA and −2pro‐PSA outperforms single free or total PSA or 2pro‐PSA. Although, PHI has a moderate diagnostic accuracy for prostate cancer, this application of this index could avoid unnecessary biopsy and treatment.

The main strength of the present is that we strictly followed PRISMA guidelines to perform this meta‐analysis and the quality of the included studies is quite high. Furthermore, the total sample size is more than 5000 patients and provides better estimations. Several study limitations need to be addressed. First, the Q test and *I* ^2^ indicated that the heterogeneity within studies is high. The subgroup analysis indicated the sample size and ethnicity may be the sources of heterogeneity. But the changes in heterogeneity are limited. This effect may be in statistical level. Besides, larger sample size means that the results tend to be more accurate. But this result needs to be confirmed in other studies. Some other potential factors cannot be further assessed because of the data unavailability. Second, there are several studies with different cut‐off values. But the sensitivity analysis indicated no potential significant differences in the diagnostic ability. Third, some studies did not provide the qualitative data; we obtained these estimated results from receiver operating characteristic, which may affect the pooled results. Finally, the present study put some search restriction in Chinese and English, other studies published in other language and gray documents may influence the estimated results. Further research is needed.

In conclusion, the PHI has a moderate accuracy for detecting prostate cancer. The diagnostic accuracy of PHI is slightly prior to free/total PSA. The ethnicity seems to have an influence on the diagnostic ability of PHI. Based on these findings, different diagnostic threshold value should be set in different ethnicity. Studies with larger sample sizes and strict design are needed to confirm the present findings. Besides, combined diagnosis with other parameters should be recommended because combined diagnosis may improve the diagnostic accuracy.
