# Supplementary material for: Assessment on clinical value of prostate health index in the diagnosis of prostate cancer
Source: Cancer Med. 2019 Jul 17;8(11):5089–96. doi: 10.1002/cam4.2376 (PMC6718540; doi:10.1002/cam4.2376)
Supplement: Supplementary file 2 [file CAM4-8-5089-s002.docx]

**Table S1** General characteristics of included study in the meta-analysis

| **Author** | **Year** | **Country** | **Study design** | **Age** | **Gold standard** | **PHI value** | **Sample size** | **TP** | **FP** | **FN** | **TN** | **Sensitivity** | **Specificity** |
| --- | --- | --- | --- | --- | --- | --- | --- | --- | --- | --- | --- | --- | --- |
| Abrate | 2015 | Span | Retrospective | >45 | Biopsy | 53.0 | 142 | 52 | 16 | 13 | 40 | 0.80 | 0.71 |
| Catalona | 2011 | USA | Prospective | >50 | Biopsy | 37.5 | 892 | 258 | 137 | 172 | 325 | 0.60 | 0.70 |
| Chiu | 2016 | China | Prospective | 65.5 | Biopsy | 35 | 267 | 101 | 30 | 32 | 104 | 0.76 | 0.78 |
| Fossati | 2015 | Italy | Retrospective | 54.7 | Biopsy | 41.2 | 238 | 42 | 64 | 25 | 107 | 0.63 | 0.63 |
| Furuya | 2017 | Japan | Prospective | 68.5 | Biopsy | 45.5 | 50 | 21 | 4 | 12 | 13 | 0.64 | 0.76 |
| Guazzoni | 2011 | Italy | Prospective | 63.3 | Biopsy | 48.5 | 268 | 96 | 92 | 11 | 69 | 0.90 | 0.43 |
| Houlgatte | 2012 | French | Prospective | 65.2 | Biopsy | 40.3 | 451 | 146 | 42 | 97 | 166 | 0.60 | 0.80 |
| Lzaaeri | 2013 | Italy | Prospective | >50 | Biopsy | 40.3 | 158 | 46 | 25 | 25 | 62 | 0.65 | 0.71 |
| Lazzeri | 2016 | Italy | Prospective | 67.3 | Biopsy | 63.9 | 262 | 92 | 36 | 34 | 100 | 0.73 | 0.74 |
| Le | 2010 | USA | Prospective | 65 | Biopsy | - | 63 | 23 | 13 | 3 | 24 | 0.88 | 0.65 |
| Loeb | 2015 | USA | Prospective | 63 | Biopsy | - | 658 | 227 | 120 | 97 | 214 | 0.70 | 0.64 |
| Mearini | 2014 | Italy | Prospective | 65.4 | Biopsy | 49.9 | 275 | 61 | 55 | 25 | 134 | 0.71 | 0.71 |
| Ng | 2014 | China | Retrospective | 65.9 | Biopsy | - | 230 | 17 | 63 | 4 | 146 | 0.81 | 0.70 |
| Perdona | 2013 | Italy | Prospective | 64.9 | Biopsy | 43.8 | 160 | 34 | 39 | 13 | 74 | 0.72 | 0.65 |
| Tan | 2016 | China | Prospective | 65.4 | Biopsy | 55.0 | 157 | 27 | 53 | 3 | 104 | 0.90 | 0.66 |
| Yu | 2016 | China | Retrospective | 67 | Biopsy | - | 261 | 78 | 33 | 19 | 131 | 0.80 | 0.80 |
| Ferro | 2013 | Italy | Prospective | 60 | Biopsy | - | 300 | 82 | 46 | 26 | 146 | 0.76 | 0.76 |
| Hu | 2017 | China | Retrospective | 71.5 | Biopsy | 42.16 | 210 | 46 | 55 | 7 | 102 | 0.87 | 0.65 |
| Wu | 2015 | China | Prospective | - | Biopsy | - | 101 | 12 | 29 | 4 | 56 | 0.75 | 0.66 |
| Sun | 2018 | China | Prospective | 68 | Biopsy | - | 391 | 132 | 62 | 43 | 154 | 0.75 | 0.71 |
